# Alterations of Brain Structural Network Connectivity in Type 2 Diabetes Mellitus Patients With Mild Cognitive Impairment

**DOI:** 10.3389/fnagi.2020.615048

**Published:** 2021-02-04

**Authors:** Chang Li, Jingna Zhang, Mingguo Qiu, Kaijun Liu, Yang Li, Zhiwei Zuo, Xuntao Yin, Yuqi Lai, Jingqin Fang, Haipeng Tong, Yu Guo, Jian Wang, Xiao Chen, Kunlin Xiong

**Affiliations:** ^1^Department of Radiology, Daping Hospital, Army Medical University, Chongqing, China; ^2^Department of Radiology, Southwest Hospital, Army Medical University, Chongqing, China; ^3^Department of Medical Imaging, College of Biomedical Engineering, Army Medical University, Chongqing, China; ^4^Department of Gastroenterology, Daping Hospital, Army Medical University, Chongqing, China; ^5^Department of Radiology, General Hospital of Western Theater Command, Chengdu, China; ^6^Department of Medical Imaging, Guizhou Provincial People's Hospital, Guiyang, China; ^7^School of Foreign Languages and Cultures, Chongqing University, Chongqing, China; ^8^Chongqing Clinical Research Center for Imaging and Nuclear Medicine, Chongqing, China; ^9^Department of Nuclear Medicine, Daping Hospital, Army Medical University, Chongqing, China

**Keywords:** type 2 diabetes mellitus, mild cognitive impairment, diffusion tensor imaging, white matter network, network-based statistics

## Abstract

Patients with type 2 diabetes mellitus (T2DM) are highly susceptible to developing dementia, especially for those with mild cognitive impairment (MCI), but its underlying cause is still unclear. This study aims to investigate the early detection of white matter structural network changes in T2DM patients with MCI and assess the relationship between cognitive impairment and structural network alterations in T2DM patients. In this study, we performed a battery of neuropsychological tests and diffusion tensor MRI in 30 T2MD-MCI patients, 30 T2DM patients with normal cognition (T2DM-NC) and 30 age-, sex-, and education-matched healthy control (HC) individuals. Cognitive performance exhibited obvious differences among the three groups. The structural network was significantly disrupted in both global and regional levels in T2DM patients. The T2DM-MCI group showed more severe impairment of global network efficiency, and lower nodal efficiency and fewer connections within multiple regions like the limbic system, basal ganglia, and several cortical structures. Moreover, a subnetwork impaired in T2DM-MCI patients was characterized by cortical-limbic fibers, and commissural fibers and pathways within the frontal, temporal, and occipital lobes. These altered global and nodal parameters were significantly correlated with cognitive function in T2DM-MCI patients. In particular, executive dysfunction and working memory impairment in T2DM-MCI patients correlated with nodal efficiency in the right opercular part and triangular part of the inferior frontal gyrus, which indicated that white matter disruption in these regions may act as potential biomarkers for T2DM-associated MCI detection. Our investigation provides a novel insight into the neuropathological effects of white matter network disruption on cognition impairments induced by T2DM.

## Introduction

Type 2 diabetes mellitus (T2DM) is a common metabolic disorder characterized by insulin resistance and hyperglycemia, which can lead to severe multi-systemic impairments. Patients with T2DM have a considerably high risk of developing cognitive dysfunction (Cukierman et al., 2005; Koekkoek et al., 2015). A significant portion of T2DM patients with cognitive impairment eventually progress to dementia (Cheng et al., 2012; Ninomiya, 2014). Mild cognitive impairment (MCI) is the early stage of diabetic cognitive impairment, which is a modifiable stage between normal cognitive aging and dementia (Sperling et al., 2011). The occurrence of dementia is an irreversible process making the pre-dementia stage, which includes MCI, the best time to treat T2DM patients (Mitchell and Shiri-Feshki, 2008). Therefore, early identification and detection of alterations in T2DM-MCI patients may help clinicians with early prevention of severe cognitive decline.

Previous studies have reported that changes in white matter (WM) in T2DM patients are correlated with cognitive impairment (Reijmer et al., 2013a; Hoogenboom et al., 2014; Zhang et al., 2014). Diffusion tensor imaging (DTI), a sophisticated magnetic resonance (MR) imaging technique, is sensitive in detecting microstructural alterations of the WM in T2DM (Hsu et al., 2012; Tan et al., 2016). These results revealed that significant alterations of WM integrity were predominantly found in the cingulum, the uncinate fasciculus, the superior and inferior longitudinal fasciculus, corpus callosum, and external and internal capsule in T2DM patients (Reijmer et al., 2013a; Hoogenboom et al., 2014; Zhang et al., 2014; Nouwen et al., 2017; Yoon et al., 2017; Sun et al., 2018; Xiong et al., 2019). However, these previous studies mainly focused on the regional abnormalities in WM integrity in T2DM.

The human brain is a complex network which comprises multiple brain regions connected to each other. Recently, there has been much interest in mapping the “connectome” to investigate and fully understand the complexity of brain structural network connections (Sporns et al., 2005; Lichtman et al., 2008). So far, four studies have examined the topological properties of the structural brain network in patients with diabetes (Reijmer et al., 2013b; Kim et al., 2016; Zhang et al., 2016, 2019). The results showed disruptions in network integration parameters which represent the slower speed and lowered capacity of a network to exchange information. However, indicators of small-world alteration and local information integration capability did not show a consistent pattern in T2DM. The reason for the inconsistent results may be that the T2DM patients studied were in different stages of diabetes-associated cognitive impairment in this research. Therefore, our study aimed to explore the WM structural network connectome mechanism in T2DM patients with and without MCI.

In this study, we enrolled T2DM patients with MCI (T2DM-MCI), T2DM patients with normal cognition (T2DM-NC), and healthy controls (HC) to perform a clinical assessment, a battery of neuropsychological tests, and DTI MR imaging. Network-based statistics (NBS) and graph theory analysis were conducted to investigate WM structural network connectome differences in these three groups, and then we assessed the relationship between structural topological network disruptions and cognitive performance in T2DM-MCI patients. Our study may provide insights into the neuropathological mechanisms of T2DM-related cognitive impairment.

## Materials and Methods

### Participants

Thirty patients with T2DM-NC and 30 patients with T2DM-MCI were enrolled in the study from October 2015 to November 2019. T2DM was diagnosed using the 1999 criteria proposed by the World Health Organization. The diagnosis of MCI was based on the criteria established in the 2006 European Alzheimer's Disease Consortium, which includes complaints of hypomnesis, an MoCA score <26, a mini mental state exam (MMSE) score > 24, a clinical dementia rating (CDR) ≥ 0.5, and a normal activities of daily living (ADL) score. Participants were excluded if they had a history of brain injury, alcoholism, epilepsy, Parkinson disease, major depression, or other psychiatric or neurological disorders. Participants with dementia (MMSE ≤ 24), severe depression (Hamilton Depression Rating Scale ≥ 18), severe claustrophobia, or contraindications to MRI were also excluded. T2DM patients were excluded if they had microvascular complications, including nephropathy, retinopathy, and neuropathy. Thirty volunteers without vascular risk factors, nervous system diseases, cognitive complaints, or psychiatric illnesses were recruited as HC. Thirty HCs had no T2DM, psychiatric or neurological disorder, and had an MoCA score ≥ 26, and an MMSE score > 24. Height, weight, and body mass index (BMI) were measured for each participant. All participants were tested by neurological, neuropsychological, and structural MRI examinations. All participants were right-handed and native Chinese speakers. Glycosylated hemoglobin (HbA1c), fasting plasma glucose (FPG), fasting C-peptide, fasting insulin, triglycerides (TGs), total cholesterol (TC), low-density lipoprotein (LDL), high-density lipoprotein (HDL), homocysteine, urinary microalbumin, blood urea nitrogen (BUN), uric acid, serum creatine, cystatin C, free thyroxine (FT), free triiodothyronine (FT3), and thyroid-stimulating hormone (TSH) were measured via standard laboratory testing. This study was carried out in accordance with the Declaration of Helsinki. The Research Ethics Committee of Southwest Hospital, Army Medical University approved the study. All participants voluntarily provided written informed consent.

### Neuropsychological Testing

All participants completed a battery of neuropsychological tests, including MMSE, Montreal Cognitive Assessment (MoCA), Digit Symbol Coding Test (DSCT), Complex Figure Test (CFT), Trail-Making Test (TMT), Digit Span Test (DST), Verbal Fluency Test (VFT), and Auditory Verbal Learning Tests (AVLT) which contains 12 variants, including coat, trousers, kerchief, glove, driver, carpenter, soldier, lawyer, crabapple, lily, wintersweet, and yulan.

### Imaging Acquisition

All imaging data were obtained on a 3-Tesla Trio MRI system (Siemens Healthcare, Erlangen, Germany) equipped with a 12-channel phase-array head coil. DTI images were acquired by a single-shot echo planar imaging (EPI) sequence with the following parameters: repetition time (TR) = 10,000 ms; echo time (TE) = 92 ms; flip angle = 90°; field of view (FOV) = 256 mm × 256 mm; matrix = 128 × 128; slice thickness = 2 mm, no gap; 75 axial slices; 64 encoding diffusion directions with b = 1,000 s/mm^2^; and 1 non-diffusion b = 0 s/mm^2^ images. The 3D high-resolution structural images were obtained using a T1-weighted magnetization prepared rapid acquisition gradient echo (MPRAGE) sequence, as follows: TR = 1,900 ms, TE = 2.52 ms, TI = 900 ms, flip angle (FA) = 9, matrix = 256 × 256, thickness = 1.0 mm, 176 slices with voxel size =1 × 1 × 1 mm. Then, all the subjects were required to undergo conventional brain T1-weighted and fluid attenuated inversion recovery (FLAIR) images to exclude organic diseases and white matter (WM) hyperintense lesions.

### Imaging Preprocessing

For the DTI data, they were preprocessed and analyzed by the Pipeline for Analyzing Brain Diffusion Images toolkit (PANDA, www.nitrc.org/projects/panda) (Cui et al., 2013) based on the FMRIB Software Library (FSL) (Behrens et al., 2003). The specific preprocessing steps were performed as previously reported (Xie X. et al., 2017). In brief, first, we converted the image format from DICOM to NIFTI. Second, we removed the skull and extracted the brain tissue. Third, we corrected head motion and eddy-current induced distortions by realigning each diffusion-weighted image to the non-weighted b0 image.

### Network Construction

The network nodes and edges were defined by the following procedures. An Automated Anatomical Labeling (AAL) template was used to parcellate the cerebral cortex into 90 anatomical regions (45 for each hemisphere), each representing a node of the constructed network. The 3D T1-weighted images were co-registered to the original b0 images using SPM (www.fil.ion.ucl.ac.uk/spm), and then the co-registered 3D-T1 images were normalized to the Montreal Neurologic Institute (MNI) space. Finally, the inverse transformations were applied to the AAL atlas, resulting in corresponding AAL regions in the individual DTI native-space.

A deterministic fiber assignment with the continuous tracking (FACT) algorithm was used to track the whole-brain white matter fibers for each subject in the native diffusion space. All voxels with an FA ≥0.2 were used as seed points; the tracking continued to one of the seed points unless the tracking angle between two adjacent voxels was > 45° or the FA was <0.2 (Mori et al., 1999). To determine the edges of the brain network, two brain regions were considered connected by an edge in cases in which at least three fibers were present between the regions. A threshold of three fibers ensures that influence from spurious connections is reduced (Lo et al., 2010). For each edge of the network, the mean FA along the fiber bundles were defined as the weights of a network edge between two connected nodes. FA value is an important index to evaluate fiber integrity (Beaulieu, 2002). Previous studies proposed that mean FA has the ability to subtly detect local brain lesions (Lim and Helpern, 2002). At last, a 90 × 90 matrix was generated, representing the FA-weighted structural network of each subject.

### Network Measures

The global and nodal topological metrics were calculated with the graph theoretical network analysis toolbox (GRETNA; http://www.nitrc.org/projects/gretna). A sparsity threshold range of 10–30% with an interval of 1%, which was checked by previous studies and showed good small-world characteristics, was applied to minimize the possible discrepancies among all FA-weighted matrices (Watts and Strogatz, 1998; Zhang et al., 2011; Korgaonkar et al., 2014). Within every threshold, the global network metrics were calculated. Specifically, the global metrics of each structural network were constant and ranged from 15 to 30% (Supplementary Figure 1). Hence, the threshold at different levels of sparsity, ranging from 10 to 15%, was employed in our study. During these thresholds, we calculated the following global network parameters of the weighted brain structural network (see detailed computational formulas of these parameters in the Supplementary Material): clustering coefficient (C_p_), characteristic path length (L_p_), normalized C_p_ (γ), normalized L_p_ (λ), small-worldness (σ), global efficiency (E_glob_), and local efficiency (E_loc_). C_p_ represented the average clustering coefficients of all nodes. L_p_ was defined as the average of the shortest path length between any two nodes. E_glob_ is considered as an important parameter to estimate the global transmission efficiency of the network. E_loc_ demonstrated the network interconnection among the nodes. We investigated the “small worldness” by generating 1,000 random networks to compare with the real networks. γ and λ were defined as the ratio of real world network's C_p_ and L_p_ to the random network's. And σ was defined as the ratio of γ to λ, which quantifies the organization of a network, with σ > 1 indicating a network has a small-world property. Nodal efficiency and betweenness centrality were also computed to represent regional characteristics of the structural network. Among these nodes, there were a number of specific nodes that interacted with many other nodes, which were defined as hubs that play a vital role in maintaining network stability. As a hub, the condition should be the betweenness centrality of a node greater than or equal to the average value plus SD of the network betweenness centrality.

Moreover, a network-based statistic (NBS) was used to identify the altered structural connections in patients (Zalesky et al., 2010). We first applied one-way ANOVA (*post-hoc*: two-sample two-tailed *t*-tests) to compare the strength of the edge at each individual element of the connectivity matrix. Second, a primary component-forming threshold (*P* < 0.01, uncorrected) was applied to search for potential connected edges. Third, the size of the largest remaining subthreshold connected component was computed. Then, the groups were randomly shuffled (5,000 permutations) and the largest subthreshold connected component size was calculated by repeating steps 1, 2, and 3. In this way, an empirical null distribution was generated to evaluate the statistical significance of the network connected component sizes. Finally, for any connected component of size M that found in the right grouping of controls and patients, the corrected *P-* value was determined by calculating the proportion of the 5,000 permutations for which the maximal connected component was larger than M. The NBS identifies the subnetworks of connected edges that differ the most among groups (*P* < 0.05 NBS corrected for multiple comparisons). The results of the nodal and edge comparisons were visualized using the BrainNet viewer software (http://www.nitrc.org/projects/bnv/).

### Statistical Analysis

All statistical analyses were performed in SPSS (version 22, Chicago, IL). For demographic and neuropsychological testing, a one-way analysis of variance (ANOVA) test was used to compare among groups. *Post-hoc* tests with Bonferroni correction were performed after observing statistical differences among the three groups. An χ^2^ test was used to compare sex variables. Instead of using single threshold metrics, we calculated the area under the curve (AUC) of each metric above to summarize the topological characteristic of the structural brain network. The group differences in AUC values of global network metrics (C_p_, L_p_, E_glob_, and E_loc_) and nodal properties (nodal efficiency and nodal betweenness centrality) were investigated with one-way ANOVA, while age and gender were adjusted as potential confounders. Metrics showing the main effect of group differences in the ANOVA model were further evaluated by *post-hoc* tests. A significance threshold of *P* < 0.05 was applied to each test, and the false discovery rate (FDR)-correction was applied for multiple comparison corrections. In addition, to explore the relationships between the topological properties of the network measures and clinical outcomes in cognitive function, we further performed Spearman's correlation between the topological properties and cognitive test scores in the T2DM-MCI patient groups. *P* < 0.05 was considered statistically significant.

## Results

### Demographics and Neuropsychological Testing

There were no significant differences in age, gender, years of education, systolic or diastolic blood pressure, LDL, total cholesterol, and triglycerides among the T2DM-MCI, T2DM-NC, and HC groups. Compared with HC, T2DM-NC patients had higher FPG and HbA1c and lower HDL (Bonferroni-correction, *P* < 0.05). T2DM-MCI patients had higher BMI, HbA1c, and FPG and lower HDL than HC (Bonferroni-correction, *P* < 0.05). But no significant difference was found between T2DM-NC and T2DM-MCI patients (Bonferroni-correction, *P* > 0.05). In the neuropsychological test, T2DM-MCI patients performed worst among the three groups, while no significant difference was shown between T2DM-NC patients and HC (Bonferroni-correction, *P* > 0.05). Moreover, T2DM-MCI patients presented an obvious reduction in multiple domains of cognitive function including episodic memory, executive function, working memory, and attention when compared with T2DM-NC patients and HC. However, no significant differences in spatial processing and language ability among the three groups were found. Demographic data and neuropsychological testing are presented in Table 1.

**Table 1:** Demographic and neuropsychological characteristics.

	**HC (*n* = 30)**	**T2DM without**	**T2DM with**	***F*-value**	***P-*value**
		**MCI (*n* = 30)**	**MCI (*n* = 30)**	**(t/χ^**2**^)**	
Numbers	30	30	30	—	—
Age (years)	53.17 ± 6.57	54.97 ± 5.54	55.9 ± 6.54	1.491	0.231
Sex (male/female)	14/16	19/11	12/18	3.467	0.177^a^
Education (years)	11.80 ± 2.99	11.90 ± 2.92	10.43 ± 2.94	2.316	0.105
Diabetes duration (years)	—	7.93 ± 5.98	6.93 ± 5.46	0.699	0.933
BMI (kg/m^2^)	23.45 ± 2.59	24.08 ± 3.02	25.59 ± 3.31	4.052	0.021^*^
Systolic blood pressure (mmHg)	128.13 ± 18.10	131.37 ± 14.78	130.57 ± 18.25	0.194	0.749
Diastolic blood pressure (mmHg)	79.77 ± 9.17	82.90 ± 9.80	80.60 ± 10.33	0.895	0.441
**Biochemical indicator**					
Fasting glucose (mmol/L)	5.47 ± 0.63	8.56 ± 1.97	9.14 ± 3.05	25.769	<0.001^*^^#^
HbA1C (%)	5.50 ± 0.36	8.84 ± 1.72	9.13 ± 2.07	49.505	<0.001^*^^#^
Total cholesterol (mmol/L)	5.21 ± 0.96	4.96 ± 1.37	5.24 ± 1.45	0.432	0.651
HDL cholesterol (mmol/L)	1.40 ± 0.33	1.18 ± 0.30	1.16 ± 0.33	5.167	0.008^*^^#^
LDL cholesterol (mmol/L)	3.10 ± 0.67	2.93 ± 0.78	3.36 ± 1.06	1.874	0.160
**General mental status**					
MoCA	27.77 ± 1.28	27.00 ± 0.83	22.93 ± 1.95	99.370	<0.001^*^^ξ^
MMSE	28.43 ± 1.17	28.43 ± 1.07	27.93 ± 1.31	1.774	0.176
**Episodic memory**					
AVLT-immediately recall	22.83 ± 5.00	22.77 ± 3.83	19.50 ± 4.55	5.406	0.006^*^^ξ^
AVLT-recognition	22.90 ± 1.45	21.87 ± 1.48	21.10 ± 3.60	4.252	0.017^*^
AVLT-delayed recall (5 min)	8.20 ± 1.94	7.80 ± 1.56	7.23 ± 2.40	1.775	0.176
AVLT-delayed recall (20 min)	7.87 ± 2.18	7.33 ± 1.77	6.83 ± 2.64	1.620	0.204
AVLT-classification	7.47 ± 2.60	7.13 ± 2.06	6.33 ± 2.25	1.902	0.155
ROCF-immediate recall	24.08 ± 8.49	22.63 ± 6.95	19.00 ± 6.64	3.754	0.027^*^
ROCF-delayed recall (20 min)	23.65 ± 7.74	21.85 ± 7.13	18.20 ± 6.19	4.659	0.012^*^
**Working memory**					
WAIS	46.23 ± 11.99	41.23 ± 10.83	35.63 ± 8.33	7.658	0.001^*^
DST-forwards	9.57 ± 1.38	8.87 ± 0.82	8.93 ± 1.08	3.584	0.032^*^
DST-backwards	5.53 ± 1.17	5.03 ± 0.89	4.67 ± 0.88	5.805	0.004^*^
**Spatial processing**					
ROCF-copy	33.17 ± 1.90	32.55 ± 4.11	31.97 ± 3.88	0.911	0.406
**Executive function**					
TMT-B	61.33 ± 22.74	67.73 ± 24.19	80.77 ± 26.59	4.877	0.010^*^
**Language ability**					
VFT	44.83 ± 6.06	44.10 ± 8.29	40.60 ± 7.04	2.970	0.057
**Attention**					
TMT-A	48.57 ± 16.61	51.97 ± 17.44	63.80 ± 21.66	5.484	0.006^*^^ξ^

All subjects (T2DM-MCI, T2DM-NC, HC) were matched for age, sex, and education. Values are the mean ± standard deviation.

a Chi-square test for sex. The comparisons of each neuropsychological test among these three groups were performed with ANOVA. The level of significance for intergroup differences was set at P < 0.05.

* P < 0.05 T2DM-MCI vs. HC with post-hoc test, Bonferroni corrected.

# P < 0.05 T2DM-NC vs. HC with post-hoc test, Bonferroni corrected.

ξ *P < 0.05 T2DM-MCI vs. T2DM-NC with post-hoc test, Bonferroni corrected. BMI, body mass index; HbA1c, glycosylated hemoglobin; HDL, high-density lipoprotein; LDL, low-density lipoprotein; MMSE, Mini-Mental State Examination; MoCA, Montreal Cognitive Assessment; AVLT, Auditory Verbal Learning Test; ROCF, Rey-Osterrieth Complex Figure; WAIS, Wechsler Adult Intelligence Scale; DST, Digital Span Test; TMT, Trail Making Test; VFT, Verbal Fluency Test*.

### Global Network Properties

**Figure 1:**
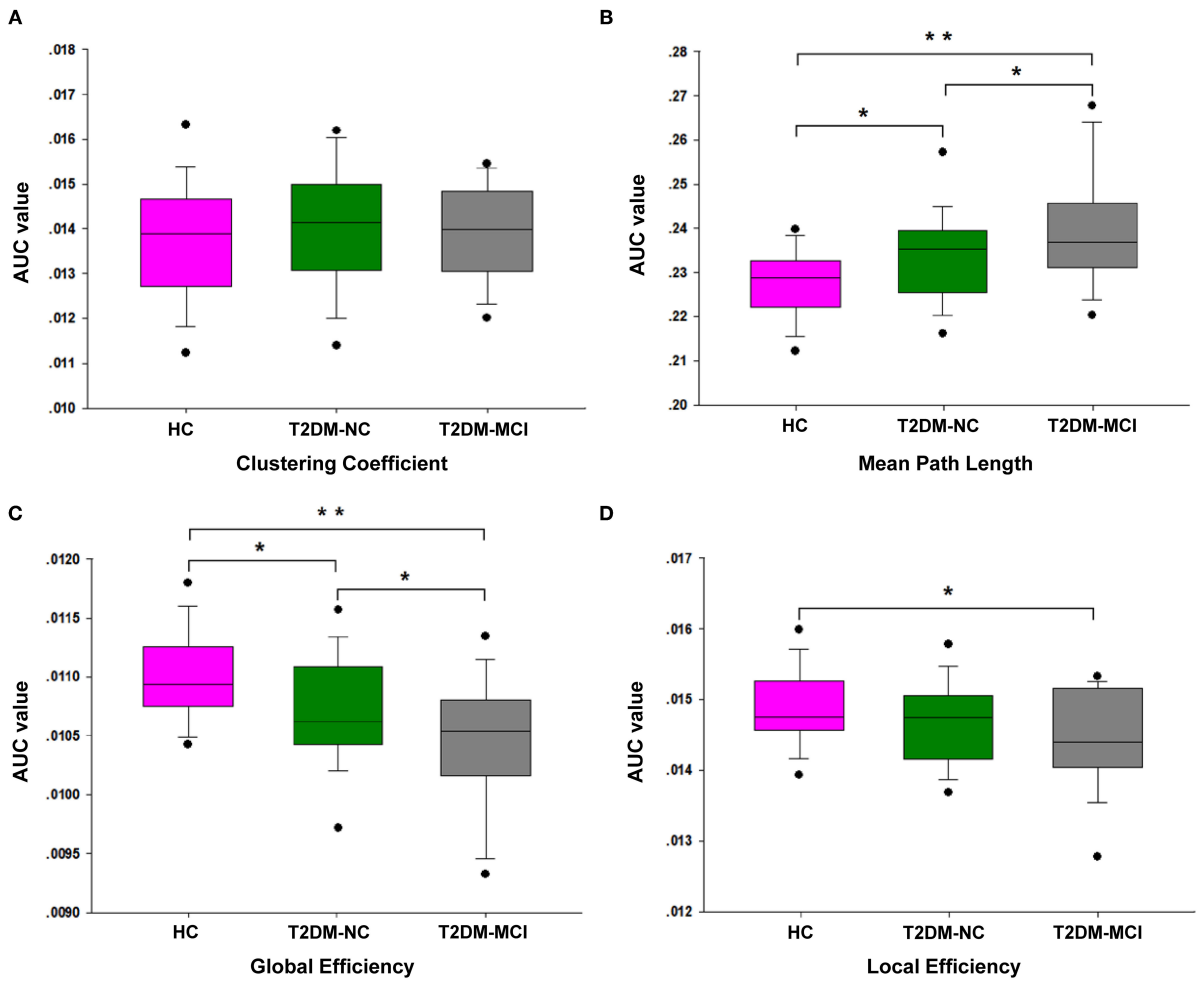
The differences of global network properties. The differences are shown in C_p_ **(A)**, L_p_ **(B)**, E_glob_ **(C)**, and E_loc_ **(D)** among the T2DM-MCI, T2DM-NC, and HC groups. *The significance threshold was set at *P* < 0.05, **The significance threshold was set at *P* < 0.001. C_p_, clustering coefficient; L_p_, shortest path length; E_glob_, global efficiency; E_loc_, local efficiency.

The brain networks of the T2DM-MCI, T2DM-NC, and HC groups exhibited good small-world properties (σ > 1, Supplementary Figure 2). The global properties of the network showed no significant differences in C_p_, but was observed in L_p_, E_glob_, and E_loc_ among these three groups. In *post-hoc* comparisons, significantly higher L_p_ and lower E_glob_ and E_loc_ were found in the T2DM-MCI group relative to the HC group, while higher L_p_ and lower E_glob_ in the T2DM-NC group were found relative to the HC group. The T2DM-MCI group showed higher L_p_ and lower E_glob_ relative to the T2DM-NC group (*P* < 0.05, FDR-corrected, Figure 1, Table 2).

**Table 2:** Differences in the global network properties among the T2DM-MCI, T2DM-NC, and HC groups.

	**HC**	**T2DM-NC**	**T2DM-MCI**	***F*-value (*P*-value)**
C_p_	0.0138 ± 0.0014	0.0141 ± 0.0014	0.0140 ± 0.0011	ns
L_p_	0.2277 ± 0.0075	0.2338 ± 0.0103	0.2399 ± 0.0128	9.618 (<0.001)
E_g_	0.0110 ± 0.0004	0.0107 ± 0.0005	0.0105 ± 0.0005	9.645 (<0.001)
E_loc_	0.0149 ± 0.0006	0.0147 ± 0.0006	0.0144 ± 0.0007	3.543 (0.033)

*ns, non-significant; C_p_, clustering coefficient; L_p_, shortest path length; E_glob_, global efficiency; E_loc_, local efficiency*.

### Nodal Efficiency and Hubs Characteristics

#### Nodal Efficiency

**Figure 2:**
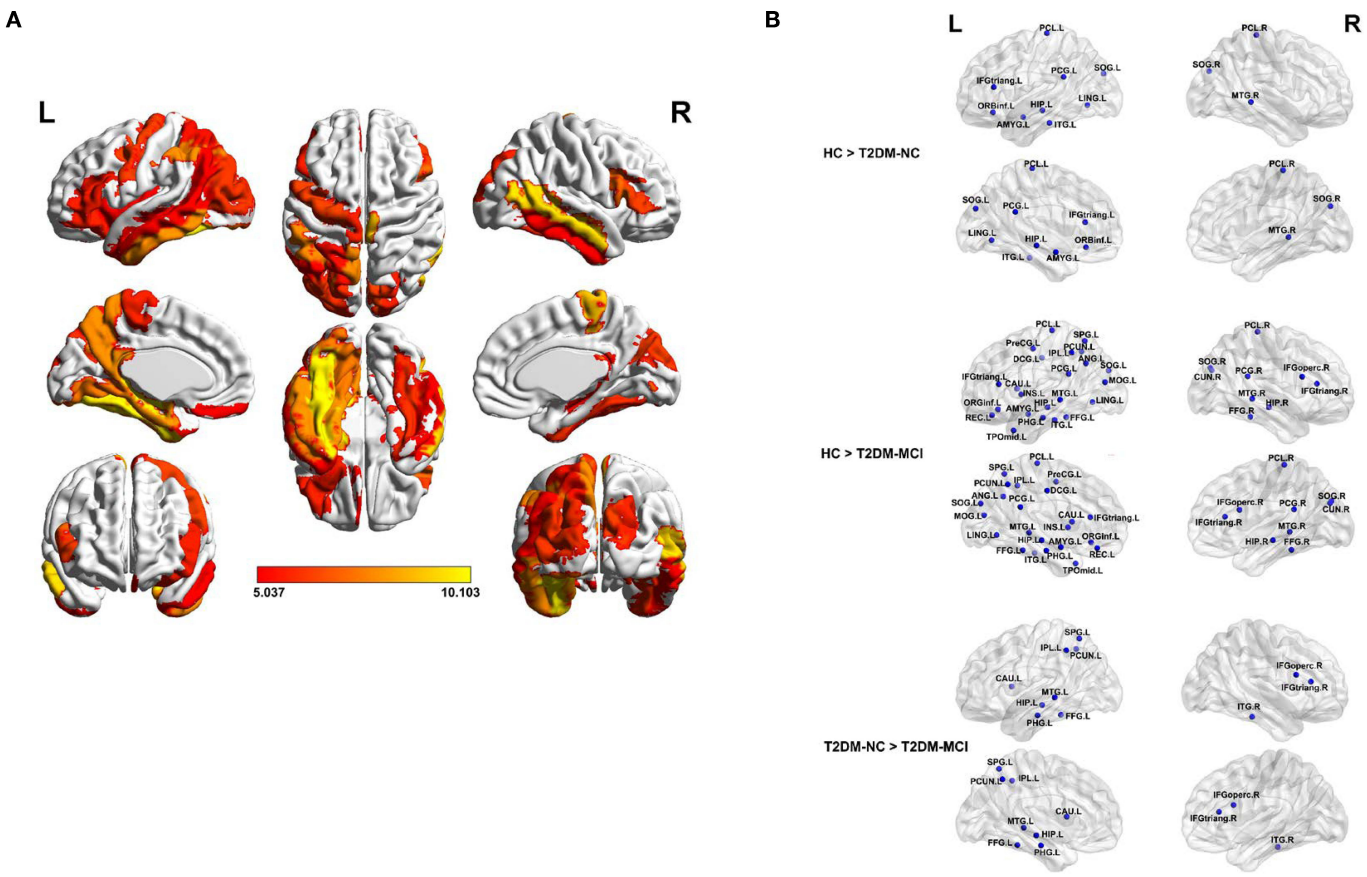
The brain regions with a significantly reduced nodal efficiency in T2DM patients. **(A)** Significant difference in terms of nodal efficiency between groups as determined by one-way ANOVA. Significant brain network nodes are rendered on the surface of the automated anatomical labeling atlas in BrainNet Viewer (NKLCNL, Beijing Normal University). The color code represents *F*-value. **(B)** Significant reductions of the nodal efficiency of T2DM-NC relative to HC (up), T2DM-MCI relative to HC (middle), and T2DM-MCI relative to T2DM-NC (bottom) were visualized. The abbreviations of the 90 brain regions are given in the Supplementary Table 1. The significance threshold was set at *P* < 0.05 (FDR-corrected). R (L) right (left) hemisphere.

All nodal properties (nodal efficiency and betweenness centrality) of the white matter network of the T2DM-MCI, T2DM-NC, and HC groups were analyzed. Significant group effects of nodal efficiency were found in 33 of 90 nodes among the three groups, while the *post-hoc* comparison showed that nodal efficiency was widely reduced at 32 nodes in the T2DM-MCI group compared with the HC group and 12 nodes in the T2DM-NC group compared with the HC group (*P* < 0.05, FDR-corrected, Table 3, Figure 2). To reveal regions specifically related to cognitive impairments associated with T2DM, we carried out a *post hoc* comparison between T2DM-MCI and T2DM-NC. Compared to T2DM-NC group, T2DM-MCI group showed lower nodal efficiency in 11 nodes mainly located in the limbic system, basal ganglia, parts of frontal, temporal and parietal lobe, including right inferior frontal gyrus (opercular part and triangular part), left hippocampus, parahippocampal gyrus, fusiform gyrus, superior parietal gyrus, precuneus, caudate nucleus, middle temporal gyrus, inferior temporal gyrus, inferior parietal, but supramarginal and angular gyri (*P* < 0.05, FDR-corrected, Table 3). There were also group effects and between-group differences of betweenness centrality in 5 of 90 nodes (*P* < 0.05). However, the differences did not survive after FDR correction for multiple comparisons (Supplementary Table 2).

**Table 3:** Differences in the nodal efficiency among the T2DM-MCI, T2DM-NC, and HC groups.

**Nodes**	**ANOVA**	***Post-hoc*** **test (*****p*****-value)**			**Nodal efficiency difference**
	**(*p*-value)**	**HC vs. T2DM-NC**	**HC vs. T2DM-MCI**	**T2DM-MCI vs. T2DM-NC**	
PreCG.L	0.009	ns	0.003	ns	HC > T2DM-MCI
IFGoperc.R	0.003	ns	0.001	0.021	HC, T2DM-NC > T2DM-MCI
IFGtriang.L	0.020	0.039	0.005	ns	HC > T2DM-NC, T2DM-MCI
IFGtriang.R	0.010	ns	0.003	0.015	HC, T2DM-NC > T2DM-MCI
ORBinf.L	0.020	0.020	0.020	ns	HC > T2DM-NC, T2DM-MCI
REC.L	0.042	ns	0.026	ns	HC > T2DM-MCI
INS.L	0.042	ns	0.012	ns	HC > T2DM-MCI
DCG.L	0.047	ns	0.023	ns	HC > T2DM-MCI
PCG.L	0.001	0.017	0.001	ns	HC > T2DM-NC, T2DM-MCI
PCG.R	0.046	ns	0.031	ns	HC > T2DM-MCI
HIP.L	<0.001	0.040	<0.001	0.009	HC > T2DM-NC > T2DM-MCI
HIP.R	0.034	ns	0.013	ns	HC > T2DM-MCI
PHG.L	0.001	ns	0.001	0.023	HC, T2DM-NC > T2DM-MC
AMYG.L	0.001	<0.001	0.007	ns	HC > T2DM-NC, T2DM-MCI
CUN.R	0.009	ns	0.002	ns	HC > T2DM-MCI
LING.L	0.002	0.014	<0.001	ns	HC > T2DM-NC, T2DM-MCI
SOG.L	0.013	0.013	0.009	ns	HC > T2DM-NC, T2DM-MCI
SOG.R	0.018	0.031	0.007	ns	HC > T2DM-NC, T2DM-MCI
MOG.L	0.012	ns	0.002	ns	HC > T2DM-MCI
FFG.L	<0.001	ns	<0.001	0.002	HC, T2DM-NC > T2DM-MC
FFG.R	0.013	ns	0.002	ns	HC > T2DM-MCI
SPG.L	0.028	ns	0.012	0.025	HC, T2DM-NC > T2DM-MC
IPL.L	0.002	ns	<0.001	0.010	HC, T2DM-NC > T2DM-MC
ANG.L	0.040	ns	0.017	ns	HC > T2DM-MCI
PCUN.L	0.002	ns	<0.001	0.011	HC, T2DM-NC > T2DM-MC
PCL.L	0.019	0.021	0.003	ns	HC > T2DM-NC, T2DM-MCI
PCL.R	<0.001	0.002	<0.001	ns	HC > T2DM-NC, T2DM-MCI
CAU.L	0.023	ns	0.011	0.039	HC, T2DM-NC > T2DM-MC
MTG.L	0.039	ns	0.022	0.040	HC, T2DM-NC > T2DM-MC
MTG.R	<0.001	0.004	<0.001	ns	HC > T2DM-NC, T2DM-MCI
TPOmid.L	0.046	ns	0.013	ns	HC > T2DM-MCI
ITG.L	0.001	0.036	<0.001	ns	HC > T2DM-NC, T2DM-MCI
ITG.R	0.043	ns	ns	0.019	T2DM-NC > T2DM-MC

*The abbreviations of the 90 brain regions are given in the Supplementary Table 1. The significance threshold was set at P < 0.05 (FDR-corrected). R (L) right (left) hemisphere. ns, non-significant*.

#### Hubs Characteristics

**Figure 3:**
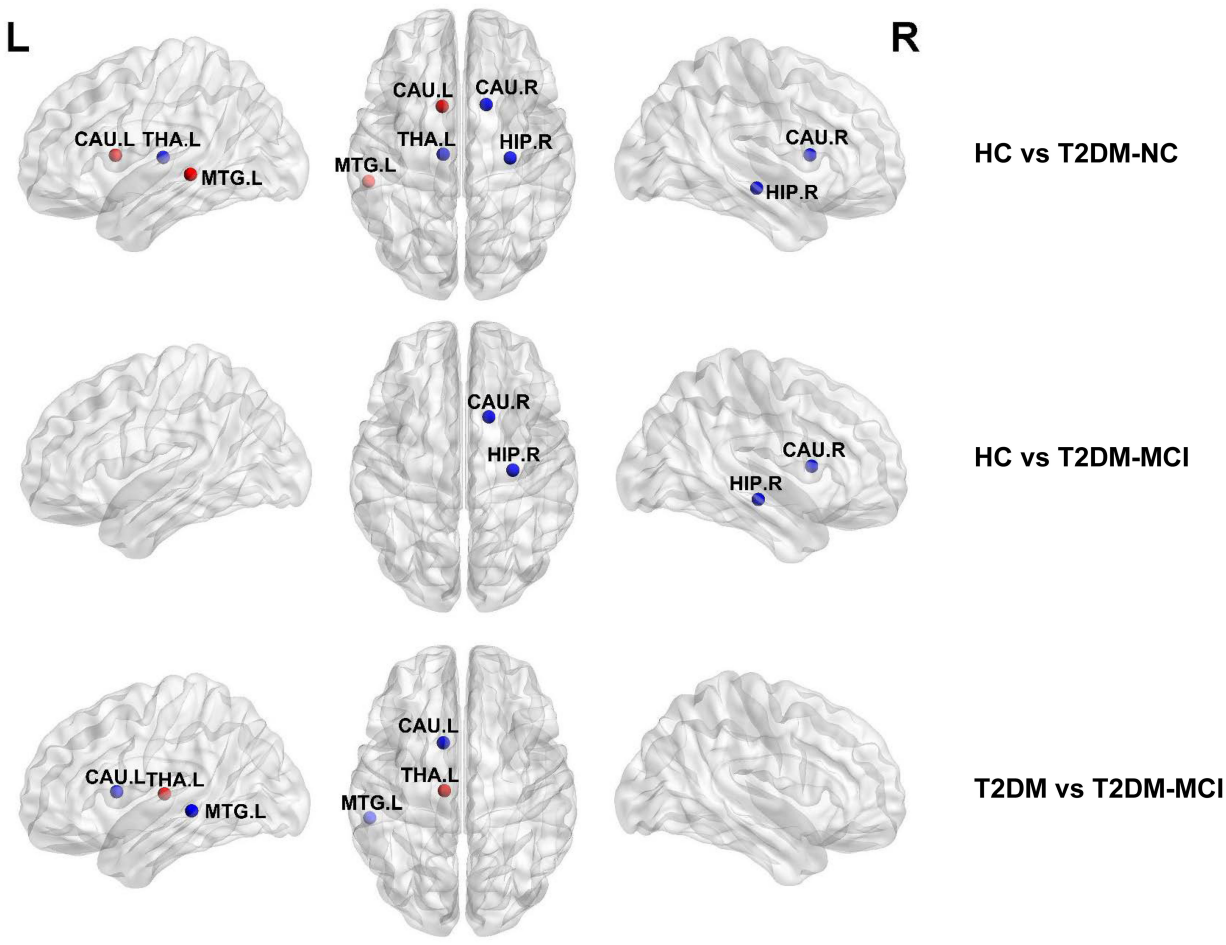
Distribution differences of hubs in the T2DM-MCI, T2DM-NC, and HC groups. The blue node represents the hub in the former group but not in the latter. In contrast, the red node represents the hub in the latter group but not in the former. HIP.R, right hippocampus; CAU.R, right caudate nucleus; CAU.L, left caudate nucleus; THA.L, left thalamus; MTG.L, middle temporal gyrus.

The hubs of the brain network of these three groups were identified, including 13 hubs in HC, 12 hubs in T2DM-NC patients, and 11 hubs in T2DM-MCI patients (Table 4). Among them, 10 hubs were shared by these three groups, mostly distributed in the bilateral precentral gyrus, calcarine fissure and surrounding cortex, superior occipital gyrus, precuneus, and putamen. Compared with HC, T2DM-NC patients had decreased hubs at the right hippocampus, right caudate nucleus, and left thalamus, while two new hubs appeared at the caudate nucleus and middle temporal gyrus on the left side (Figure 3). T2DM-MCI patients had decreased hubs at the right hippocampus and right caudate nucleus relative to HC, while they had decreased hubs at the left caudate nucleus and left middle temporal gyrus, and increased hubs at the left thalamus relative to T2DM-NC patients (Figure 3).

**Table 4:** Hubs of the T2DM-MCI, T2DM-NC, and HC groups.

**Group**	**Region**	**Group**	**Region**	**Group**	**Region**
HC	Precentral_L	T2DM-NC	Precentral_L	T2DM-MCI	Precentral_L
	Precentral_R		Precentral_R		Precentral_R
	Hippocampus_R		Calcarine_L		Calcarine_L
	Calcarine_L		Calcarine_R		Calcarine_R
	Calcarine_R		Occipital_Sup_L		Occipital_Sup_L
	Occipital_Sup_L		Occipital_Sup_R		Occipital_Sup_R
	Occipital_Sup_R		Precuneus_L		Precuneus_L
	Precuneus_L		Precuneus_R		Precuneus_R
	Precuneus_R		Caudate_L		Putamen_L
	Caudate_R		Putamen_L		Putamen_R
	Putamen_L		Putamen_R		Thalamus_L
	Putamen_R		Temporal_Mid_L		
	Thalamus_L				

*R (L) right (left) hemisphere*.

### Network Measure Correlates With Cognitive Function Score

**Figure 4:**
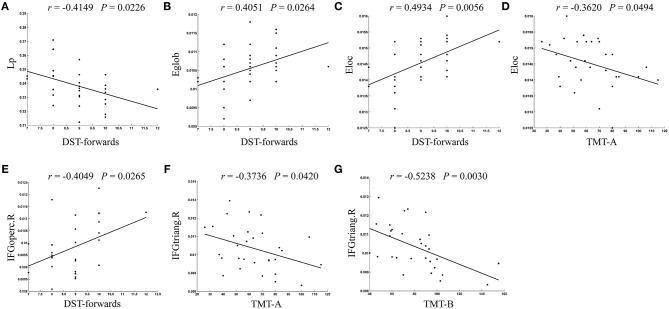
The significant correlations between altered global or nodal network properties and cognitive performance in T2DM-MCI patients. **(A)** Correlation between L_p_ and DST-forwards. **(B)** Correlation between E_glob_ and DST-forwards. **(C)** Correlation between Eloc and DST-forwards. **(D)** Correlation between E_loc_ and TMT-A. **(E)** Correlation between IFGoperc.R and DST-forwards. **(F)** Correlation between IFGtriang.R and TMT-A. **(G)** Correlation between IFGtriang.R and TMT-B. L_p_, shortest path length; E_glob_, global efficiency; E_loc_, local efficiency; IFGoperc.R, right opercular part of inferior frontal gyrus; IFGtriang.R, right triangular part of inferior frontal gyrus.

The correlations between brain network parameters, including global network properties and nodal efficiency, and cognitive function scores were analyzed for the T2DM-MCI group. For global network properties, L_p_, E_glob_, and E_loc_ were significantly correlated with DST scores (*r* = −0.4149, *P* = 0.0226; *r* = 0.4051, *P* = 0.0264; *r* = 0.4934, *P* = 0.0056, uncorrected), and E_loc_ was significantly correlated with TMT-A scores in the T2DM-MCI group (*r* = −0.3620, *P* = 0.0494, uncorrected) (Figures 4A–D). Furthermore, the nodal efficiency of the right opercular part of the inferior frontal gyrus was significantly correlated with DST scores (*r* = 0.4049, *P* = 0.0265, uncorrected) in the T2DM-MCI group (Figure 4E). And the nodal efficiency of the right triangular part of the inferior frontal gyrus was significantly correlated with TMT-A scores (*r* = −0.3736, *P* = 0.0420, uncorrected) and TMT-B scores (*r* = −0.5238, *P* = 0.00297, uncorrected), respectively (Figures 4F,G).

### Network-Based Statistical Analysis

**Figure 5:**
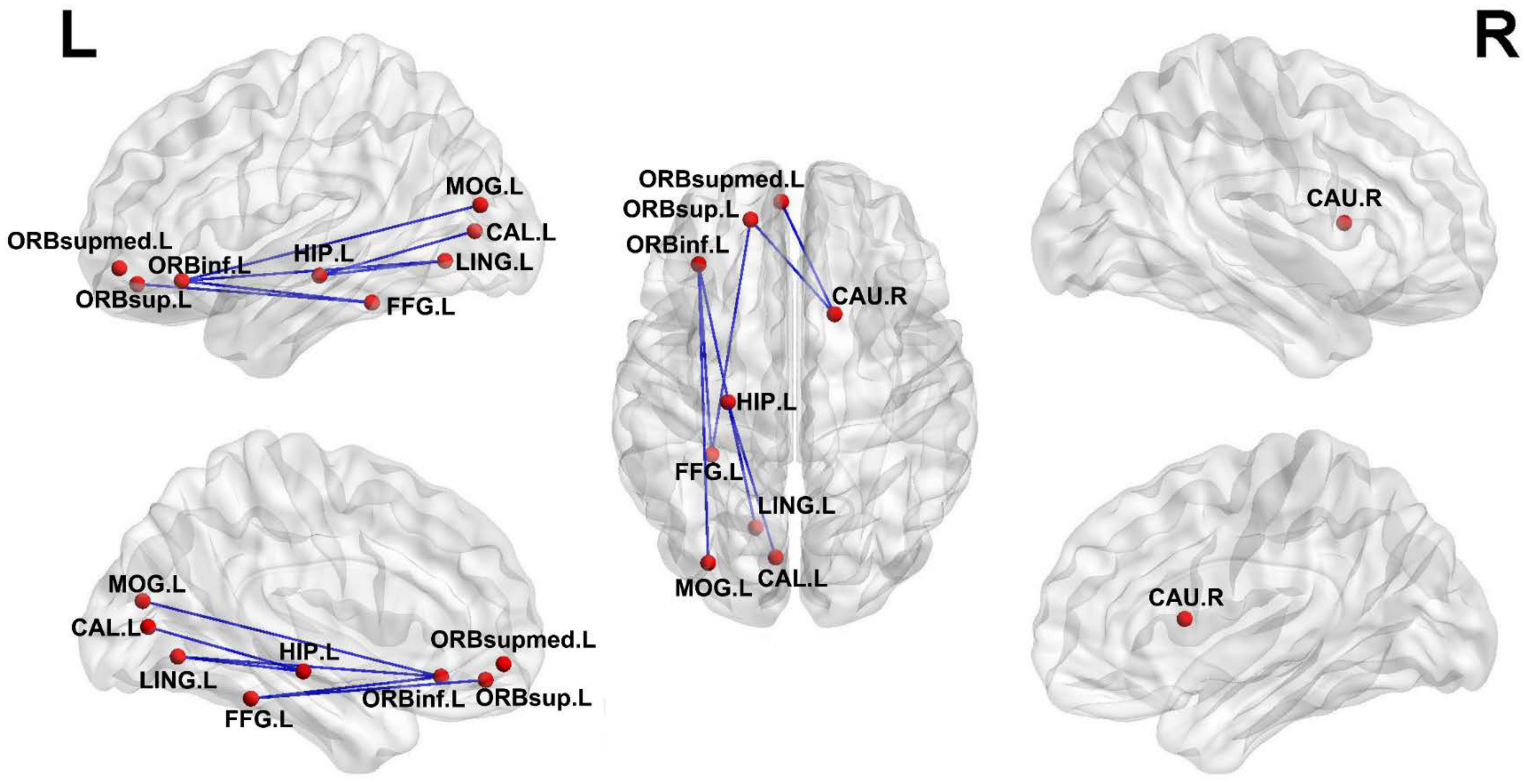
Subnetworks with impaired edge connection in T2DM-MCI patients relative to HC. The impaired connections are shown in blue edges. The nodes are set to equal size. HIP.L, left hippocampus; CAL.L, left calcarine fissure and surrounding cortex; ORBinf.L, left inferior frontal gyrus, orbital part; LING.L, left lingual gyrus; FFG.L, left fusiform gyrus; ORBsupmed.L, left superior frontal gyrus, medial orbital; CAU.R, right caudate nucleus.

Differences of the connectivity component between groups were detected using the NBS method. A subnetwork of 9 nodes and 8 edges was found impaired in the T2DM-MCI group, compared with the HC group (*P* < 0.05, NBS corrected, Figure 5). Nevertheless, no significant differences were found between the T2DM-NC group and HC group or T2DM-MCI group and T2DM-NC group. The impaired subnetwork included the left hippocampus, calcarine fissure and surrounding cortex, the orbital part of the inferior frontal gyrus, lingual gyrus, the orbital part of the superior frontal gyrus, middle occipital gyrus, fusiform gyrus, the medial orbital of the superior frontal gyrus, and right caudate nucleus.

## Discussion

The purpose of this current study was to early detect the neuroimaging alterations of T2DM-associated cognitive impairments. All participants in the T2DM-MCI, T2DM-NC, and HC groups had good small-world properties. The global properties of the structural network showed a significant decrease of E_glob_ and an increase of L_p_ in T2DM-MCI patients, when compared with T2DM-NC patients. Then nodal efficiency analysis showed the impaired efficiency of widespread brain regions in T2DM-MCI and T2DM-NC patients. While nodal efficiency, primarily located in the limbic system, basal ganglia, parts of the frontal, temporal, and parietal lobes, suffered more severely in T2DM-MCI patients than T2DM-NC. Moreover, a subnetwork impaired in T2DM-MCI was identified and characterized by cortical-limbic fibers, commissural fibers and pathways within frontal, temporal and occipital lobes. In particular, these network abnormalities were correlated with cognitive function performance in T2DM-MCI patients. Our investigations highlighted the importance of structural network analysis in our understanding of T2DM-related cognitive impairment symptoms.

In this study, deceased E_glob_ and increased L_p_ were observed in T2DM-MCI and T2DM-NC patients, which was in line with previous studies related to T2DM and cognitive impairments using DTI-based graph theoretical network analysis (Reijmer et al., 2013b; Kim et al., 2016; Zhang et al., 2016). Meanwhile, E_loc_ was also found significantly deceased in T2DM-MCI patients when compared with HC. These above findings imply that efficiency of network information transmission was decreased with the cognition decline of T2DM patients. However, no significant changes were found on C_p_ of T2DM-MCI and T2DM-NC patients in our study. The changing pattern of C_p_ reported in white matter network studies of T2DM have been controversial. Reijmer et al. found decreased C_p_ in well-controlled diabetic patients (Reijmer et al., 2013b), while Zhang et al. reported increased C_p_ in T2DM patients free of clinical vascular complications (Zhang et al., 2019). Two other studies detected no change in C_p_ in T2DM, which were similar to our current study (Kim et al., 2016; Zhang et al., 2016). These inconsistencies may be explained by the differences in sample sizes, age, and complications of the enrolled patients. Therefore, more studies are needed to draw a reliable conclusion about the impact of T2DM on the topological properties of the brain network.

T2DM-MCI patients exhibited significantly reduced nodal efficiency in 11 nodes, mainly located in the limbic system, basal ganglia, and parts of the frontal, temporal, and parietal lobes. Previous studies have reported that the temporal lobe and in particular the limbic system seem to be susceptible to T2DM (Den Heijer et al., 2003; Korf et al., 2006; Gold et al., 2007; Mccrimmon et al., 2012; Xie Y. et al., 2017). Our results are in agreement with previous literature showing microstructural and network impairment in different regions of the temporal lobe and limbic system, and also in their corresponding association fibers in T2DM. In addition, T2DM is also associated with the abnormal pattern of functional connectivity in these regions (Xia et al., 2017). These structures have been implicated in memory formation and learning (Mccrimmon et al., 2012). Furthermore, it had been well-documented that deficits in either frontal cortex or WM may be the underlying reasons for executive dysfunction and memory loss in patients with T2DM (Duncan and Owen, 2000; Preston and Eichenbaum, 2013; Reijmer et al., 2013b; Duan et al., 2015). Of note, our findings revealed that the nodal efficiency in the right opercular part and triangular part of the inferior frontal gyrus had a close relationship with multiple cognitive function scores, especially working memory, attention, and executive function, which was in line with previous studies (Li et al., 2018). The opercular part of the inferior frontal gyrus has many important functions in the brain, some of which are involved in semantic processing, language production, and phonological processing (Tonkonogy and Goodglass, 1981; Sainson et al., 2014). Moreover, the loss of white matter volume in the inferior frontal triangle region has been reported in T2DM patients (Chen et al., 2012). Both the opercular part and triangular part of the inferior frontal gyrus are involved in higher cognitive functions such as memory, emotion, and learning (Maess et al., 2006; Badre and Wagner, 2007). These results suggested that WM in the right opercular part and triangular part of the inferior frontal gyrus may act as potential biomarkers for T2DM-associated MCI detection.

More importantly, brain hubs, defined as some specific nodes interacting with many other nodes, play an important role in maintaining network stability and enabling efficient information transmission (Albert et al., 2000). The loss of hubs could affect network connection and information processing (Van Den Heuvel and Sporns, 2013). In hubs analysis, there were almost similar hub organizations in the T2DM-MCI, T2DM-NC, and HC groups, but differences still existed in the distribution of hubs among these three groups at the right hippocampus, bilateral caudate nucleus, and right middle temporal gyrus. T2DM-NC patients lost hubs at the right hippocampus and caudate nucleus, while two new hubs were found at the left caudate nucleus and middle temporal gyrus when compared to HC. These increased hub nodes may represent compensation for those lost, which may maintain the normal cognitive function in T2DM-NC patients (Liu et al., 2018; Yu et al., 2019; Wang et al., 2020). Meanwhile, T2DM-MCI patients lost hubs at the right hippocampus and caudate nucleus relative to HC, and lost hubs at the left caudate nucleus and middle temporal gyrus compared to T2DM-NC. These lost hubs are more likely to be related to cognitive performance (Den Heijer et al., 2003; Gold et al., 2007; Falvey et al., 2013; Rofey et al., 2015). Also, some regions with decreased nodal efficiency belonged to the lost hubs in T2DM-MCI. This suggests that the hub nodes, to some extent, were yet to be damaged, resulting in cognitive impairments in T2DM-MCI patients. This subsequently explains that T2DM cognitive dysfunction might disrupt the structural network and reorganize the network's hubs.

In addition, T2DM-NC patients showed disrupted topological organization of the white matter network including global network properties, nodal efficiency, and hubs, which indicates that the topological organization of structural brain networks have already been altered in T2DM-NC patients. Our results are partly in accordance with previous studies that explored changes of structural and functional brain networks in cognitively intact T2DM patients (Chen et al., 2017; Zhang et al., 2019). However, T2DM-MCI patients showed another disrupted structural network that was worse than T2DM-NC patients. These differences illustrated the severity of the disease condition. Therefore, a longitudinal study is necessary to confirm the dynamic changes in brain network topological organization in T2DM patients as the disease progresses in future.

However, our study has a few limitations. First, the current study is a cross-sectional study with a relatively small sample size, and a longitudinal study with a larger sample size is needed in future to detect the dynamic changes in structural network properties and to test whether structural network properties can predict the development of cognitive impairment in T2DM patients. Second, there was a higher BMI index in groups with T2DM than healthy controls. However, there were no significant intergroup differences in BMI between T2DM-MCI and T2DM-NC patients, and the effect of BMI on the results should be small. Last but not least, we only focused on the structural network properties and their relationship with neurophysiological tests in T2DM patients. Exploring the overlap between functional and structural connectivity in the same participants will achieve more convincing and reliable results. Thus, multimodal imaging methods are needed to investigate the structural and functional dysconnectivity in T2DM associated with cognitive deficits in the future.

## Conclusion

In conclusion, the present study revealed disrupted network measures in T2DM patients. T2DM-MCI patients showed sporadic impairments of the structural network, primarily located in the limbic system, basal ganglia, and parts of the frontal, temporal, and parietal lobes when compared with T2DM-NC patients. Moreover, these network abnormalities were correlated with cognitive function performance in T2DM-MCI patients. In particular, the executive dysfunction and working memory impairment in T2DM-MCI patients correlated with reduced nodal efficiency in the right opercular part and triangular part of the inferior frontal gyrus, which indicated that WM in these regions may act as potential biomarkers for T2DM-associated MCI detection. Our investigation provides a novel insight into the neuropathological effects of white matter network disruption on early cognition impairments induced by T2DM.

## Data Availability Statement

The original contributions presented in the study are included in the article/Supplementary Material, further inquiries can be directed to the corresponding author/s.

## Ethics Statement

The studies involving human participants were reviewed and approved by Research Ethics Committee of Southwest Hospital, Army Medical University, Chongqing, China. The patients/participants provided their written informed consent to participate in this study.

## Author Contributions

KX, XC, and JW conceived and designed the study. CL, YLi, and ZZ collected the data for MRI, clinical, and neuropsychological tests. JZ and MQ performed the network analysis. KL and HT performed statistics analysis. JF and YG prepared the figures and tables. XC and KL wrote the manuscript. XY and YLa edited the manuscript. All authors revised the manuscript, read, and approved the submitted version.

## Conflict of Interest

The authors declare that the research was conducted in the absence of any commercial or financial relationships that could be construed as a potential conflict of interest.
