# Supplementary figures and images for: Alterations of Brain Structural Network Connectivity in Type 2 Diabetes Mellitus Patients With Mild Cognitive Impairment
Source: Front Aging Neurosci. 2021 Feb 4;12:615048. doi: 10.3389/fnagi.2020.615048 (PMC7891182; doi:10.3389/fnagi.2020.615048)

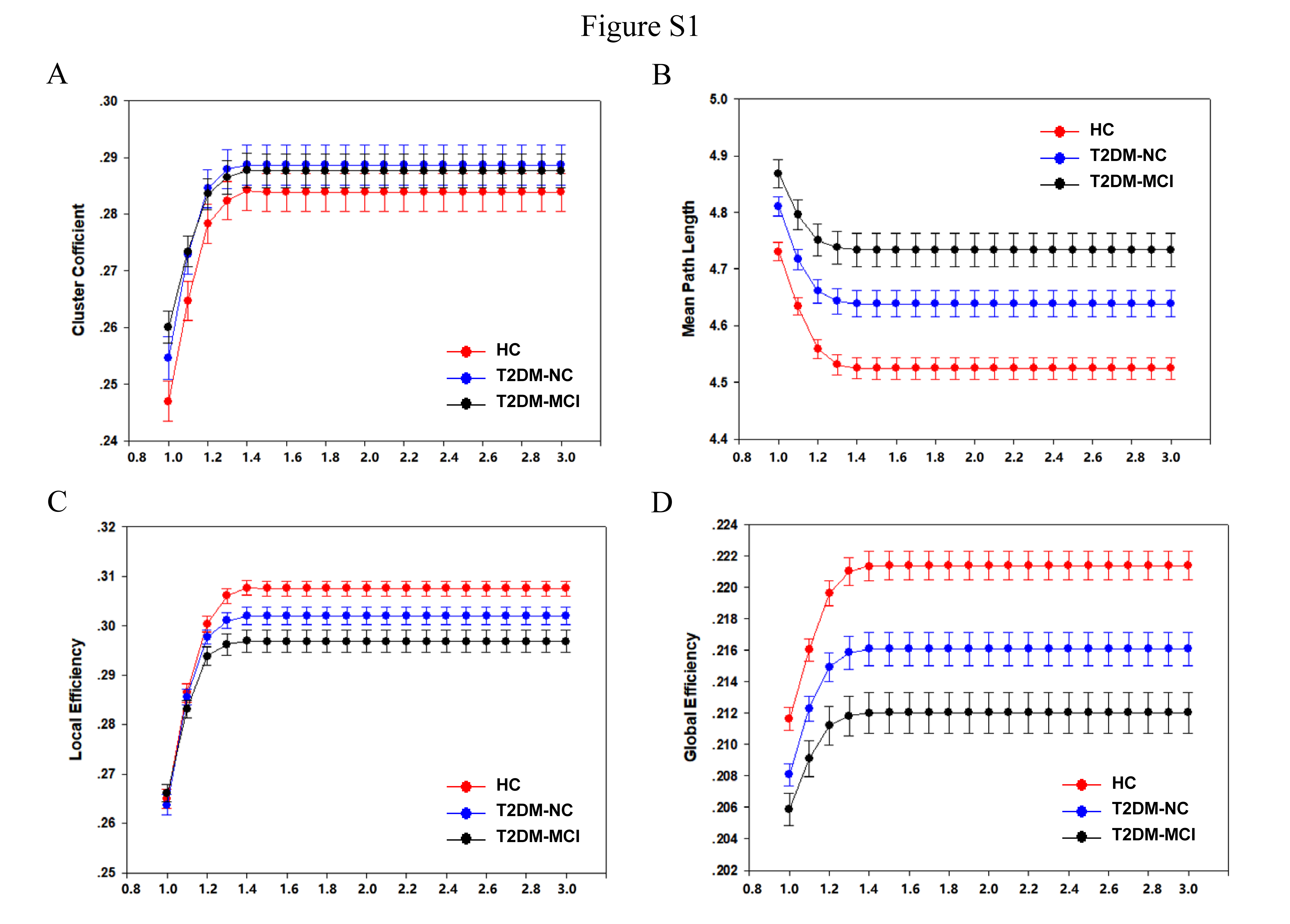

Supplement: Supplementary file 1 [file Image_1.TIFF]

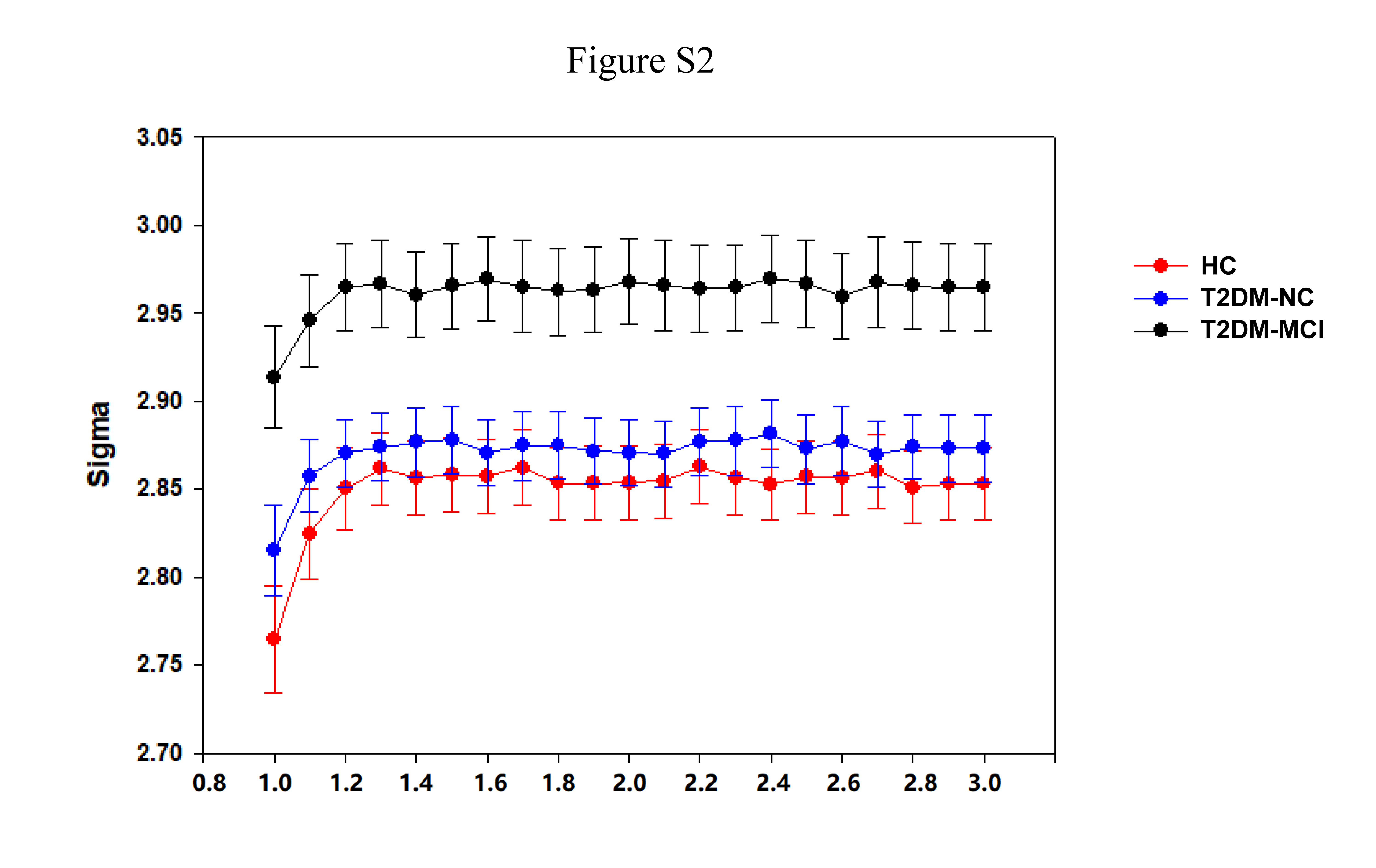

Supplement: Supplementary file 2 [file Image_2.TIFF]
