# Supplementary material for: Alterations of Brain Structural Network Connectivity in Type 2 Diabetes Mellitus Patients With Mild Cognitive Impairment
Source: Front Aging Neurosci. 2021 Feb 4;12:615048. doi: 10.3389/fnagi.2020.615048 (PMC7891182; doi:10.3389/fnagi.2020.615048)
Supplement: Supplementary file 3 [file Table_1.docx]

Supplementary Material

# Supplementary Data

**The computational formulas of global and nodal network parameters**

The weighted clustering coefficient of a node i, $C_{p}^{i}$, which was defined as the possibility whether the neighborhoods were connected with each other or not, is expressed as follows:

$$C_{p}^{i}=\frac{1}{k_{i}(k_{i}-1)} \sum_{j,h\in N} {(w_{ij}w_{ih}w_{jh})}^{1/3}$$

where $k_{i}$ is the number of edges connecting to node i, and $w_{ij}$ is the weight between node i and node j in the network. The average clustering coefficient, $C_{p}$, was expressed as the average of $C_{p}^{i}$, was expressed as the average of $C_{p}^{i}$ across all nodes in the network:

$$C_{p}=\frac{1}{N}\sum_{i\in N} C_{p}^{i}$$

where N is the number of nodes in individual network. The $C_{p}$ indicates the extent of local interconnectivity in the network.

The shortest length $L_{ij}$ is defined as the length of the path for node i and j with the shortest length. The characteristic shortest path length $L_{p}$ of a network is computed as follows:

$$L_{p}=\frac{N(N-1)}{\sum_{i=1}^{N} \sum_{j\neq i}^{N} 1/L_{ij}}$$

where N is the number of nodes. $L_{p}$ quantifies the ability for information propagation in parallel.

The global efficiency of a network, $E_{glob}$, is expressed as follows:

$$E_{glob}=\frac{1}{N(N-1)}\sum_{i\neq j\in N} \frac{1}{L_{ij}}$$

where N is the number of nodes in the network. $E_{glob}$ is a measure of parallel information transformation.

The local efficiency of a network, $E_{loc}$, is computed as follows:

$$E_{loc}=\frac{1}{N}\sum_{i\in N} E_{glob}(G_{i})$$

where $E_{glob}(G_{i})$ is the global efficiency of the neighborhood subgraph $G_{i}$ of the node i.

The regional efficiency $E_{i}$ of each node i in the weighted network was defined as the inverse of harmonic mean of the shortest path length between itself and all other nodes, which was used to quantify the importance of a node in the communication within the network.

$$E_{i}=\frac{1}{N-1}\sum_{j\in N,j\neq i} \frac{1}{L_{ij}}$$

The betweenness centrality $B_{i}$ of each node i in the weighted network, was defined as the fraction of all shortest paths in the network that pass through it, which was essentially a measurement of the influence of a node over the information flow between itself and other nodes.

$$B_{i}=\sum_{j\neq k\neq i\in N} \frac{\sigma_{jk}(i)}{\sigma_{jk}}$$

where $\sigma_{jk}\left( i \right)$ is the number of shortest path between nodes j and k that passed through node i.

| Index | Region | Abbreviations |
| --- | --- | --- |
| 1, 2 | Precentral gyrus | PreCG |
| 3, 4 | Superior frontal gyrus, dorsolateral | SFGdor |
| 5, 6 | Superior frontal gyrus, orbital part | ORBsup |
| 7, 8 | Middle frontal gyrus | MFG |
| 9, 10 | Middle frontal gyrus, orbital part | ORBmid |
| 11, 12 | Inferior frontal gyrus, opercular part | IFGoperc |
| 13, 14 | Inferior frontal gyrus, triangular part | IFGtriang |
| 15, 16 | Inferior frontal gyrus, orbital part | ORBinf |
| 17, 18 | Rolandic operculum | ROL |
| 19, 20 | Supplementary motor area | SMA |
| 21, 22 | Olfactory cortex | OLF |
| 23, 24 | Superior frontal gyrus, medial | SFGmed |
| 25, 26 | Superior frontal gyrus, medial orbital | ORBsupmed |
| 27, 28 | Gyrus rectus | REC |
| 29, 30 | Insula | INS |
| 31, 32 | Anterior cingulate and paracingulate gyri | ACG |
| 33, 34 | Median cingulate and paracingulate gyri | MCG |
| 35, 36 | Posterior cingulate gyrus | PCG |
| 37, 38 | Hippocampus | HIP |
| 39, 40 | Para hippocampal gyrus | PHG |
| 41, 42 | Amygdala | AMYG |
| 43, 44 | Calcarine fissure and surrounding cortex | CAL |
| 45, 46 | Cuneus | CUN |
| 47, 48 | Lingual gyrus | LING |
| 49, 50 | Superior occipital gyrus | SOG |
| 51, 52 | Middle occipital gyrus | MOG |
| 53, 54 | Inferior occipital gyrus | IOG |
| 55, 56 | Fusiform gyrus | FFG |
| 57, 58 | Postcentral gyrus | PoCG |
| 59, 60 | Superior parietal gyrus | SPG |
| 61, 62 | Inferior parietal lobule | IPL |
| 63, 64 | Supramarginal gyrus | SMG |
| 65, 66 | Angular gyrus | ANG |
| 67, 68 | Precuneus | PCUN |
| 69, 70 | Paracentral lobule | PCL |
| 71, 72 | Caudate | CAU |
| 73, 74 | Putamen | PUT |
| 75, 76 | Pallidum | PAL |
| 77, 78 | Thalamus | THA |
| 79, 80 | Heschl gyrus | HES |
| 81, 82 | Superior temporal gyrus | STG |
| 83, 84 | Temporal pole: superior temporal gyrus | TPOsup |
| 85, 86 | Middle temporal gyrus | MTG |
| 87, 88 | Temporal pole: middle temporal gyrus  temporal gyrus | TPOmid |
| 89, 90 | Inferior temporal | ITG |

Table S1 Abbreviations of the 90 brain regions in AAL-90 atlas

Notes: Odd and even numbers indicate brain regions of left and right hemispheres, respectively.

Table S2 Differences in the nodal betweenness centrality among T2DM-MCI, T2DM-NC and HC groups

| Nodes | ANOVA | Post hoc test (p value) | | | Nodal efficiency difference |
| --- | --- | --- | --- | --- | --- |
|  | (p value) | HC vs T2DM-NC | HC vs T2DM-MCI | T2DM-MCI vs T2DM-NC |  |
| ORBsupmed.L | 0.006 | 0.004 | ns | ns | HC > T2DM-NC |
| PCG.L | 0.049 | 0.016 | ns | ns | HC > T2DM-NC |
| PAL.L | 0.018 | ns | ns | 0.010 | T2DM-NC>T2DM-MCI |
| TPOmid.L | 0.048 | 0.025 | ns | ns | HC>T2DM-NC |
| TPOmid.R | 0.002 | 0.001 | 0.0102 | ns | HC > T2DM-NC, T2DM-MCI |

Notes: The abbreviations of the 90 brain regions are given in supplementary materials (Table S1). The significance threshold was set at p < 0.05(uncorrected). R (L) right (left) hemisphere. ns: non-significant.

**Supplementary Figure legends**

Figure S1. The global metrics of each structural network at a sparsity threshold range 10%–30% with an interval of 0.01. The global metrics of each structural network were constant and ranged from 15 to 30%.

Figure S2. Small-worldness σ increased with the sparsity thresholds range 10%–15%, and constant with the sparsity thresholds range 15%–30%. In this study, the small-world regime was defined as 10% < S < 15%, where the networks were estimable for small-worldness and individual σ > 1.
